# CRISPR/Cas9-Mediated Gene Knock-Down in Post-Mitotic Neurons

**DOI:** 10.1371/journal.pone.0105584

**Published:** 2014-08-20

**Authors:** Christoph Straub, Adam J. Granger, Jessica L. Saulnier, Bernardo L. Sabatini

**Affiliations:** Department of Neurobiology, Howard Hughes Medical Institute, Harvard Medical School, Boston, Massachusetts, United States of America; Louisiana State University Health Sciences Center, United States of America

## Abstract

The prokaryotic adaptive immune system CRISPR/Cas9 has recently been adapted for genome editing in eukaryotic cells. This technique allows for sequence-specific induction of double-strand breaks in genomic DNA of individual cells, effectively resulting in knock-out of targeted genes. It thus promises to be an ideal candidate for application in neuroscience where constitutive genetic modifications are frequently either lethal or ineffective due to adaptive changes of the brain. Here we use CRISPR/Cas9 to knock-out *Grin1*, the gene encoding the obligatory NMDA receptor subunit protein GluN1, in a sparse population of mouse pyramidal neurons. Within this genetically mosaic tissue, manipulated cells lack synaptic current mediated by NMDA-type glutamate receptors consistent with complete knock-out of the targeted gene. Our results show the first proof-of-principle demonstration of CRISPR/Cas9-mediated knock-down in neurons *in* *vivo*, where it can be a useful tool to study the function of specific proteins in neuronal circuits.

## Introduction

The typical approach to investigate the function of a given protein is to delete the genomic sequence encoding it and study the physiological effect of this ‘knock-out’ [1]. In neuroscience this approach has been used widely, but results are often limited by several technical difficulties. First, a significant subset of genes is essential for survival, and genomic deletion is lethal [2]. Second, the plasticity of the brain can lead to developmental compensation for deleted genes, thereby obscuring the effect [3]. These problems have been circumvented by generating conditional knock-out mice, in which genomic deletion of a gene depends on the presence of a recombinase whose expression can be spatially and/or temporally restricted [4]. However, this approach still requires the generation of germline genetically modified mice, a process that takes at least several months.

The difficulties of conventional transgenic mouse models could be overcome by efficient genome editing methods that would allow controlled perturbation in post-mitotic neurons. The type II prokaryotic adaptive immune system (clustered regularly interspaced short palindromic repeats (CRISPR)) [5] with the endonuclease CRISPR-associated (Cas) 9 has recently been engineered for this use in mammalian cells [6], [7]. In addition to the endonuclease Cas9, CRISPR/Cas9-mediated editing requires two short RNAs, a target-recognizing CRISPR-RNA, and a Cas9-recruiting tracer-RNA; both RNAs can be linked together as single guide-RNA (gRNA) [8]. When co-expressed with an appropriate gRNA, Cas9 is recruited to the genomic DNA in a sequence-specific manner, and cuts both strands at a precise location. The genomic DNA is then repaired by non-homologous end joining (NHEJ), introducing mutations that effectively interrupt the open reading frame, and thereby results in a functional knock-out of the encoded protein [8].

Since its first application in mammalian cells, CRISPR/Cas9 has been used in many different organisms and applications [9]–[15]; however, it is still unknown if this system can be used in mammalian neurons to generate genetically mosaic brain tissue. Here we demonstrate that CRISP/Cas9-mediated knock-down can be used to effectively delete proteins in individual post-mitotic neurons of an otherwise unperturbed brain.

## Materials and Methods

### Animal research and ethics statement

All experiments that included animals were carried out in accordance with protocols approved by the Harvard Standing Committee on Animal Care following guidelines described in the US National Institutes of Health *Guide for the Care and Use of Laboratory Animals*, and all efforts were made to minimize suffering. For *in-utero* electroporation, mice were anesthetized using 2% isoflurane and injected with 0.1 mg/kg of buprenorphrine as anesthetic. For euthanasia, mice were anesthetized with isoflurane. This study, and all procedures in it, was approved by the Harvard Medical School Institutional Animal Care and Use Committee (IACUC), protocol no. 03551.

### Cloning and constructs

**Figure 1:**
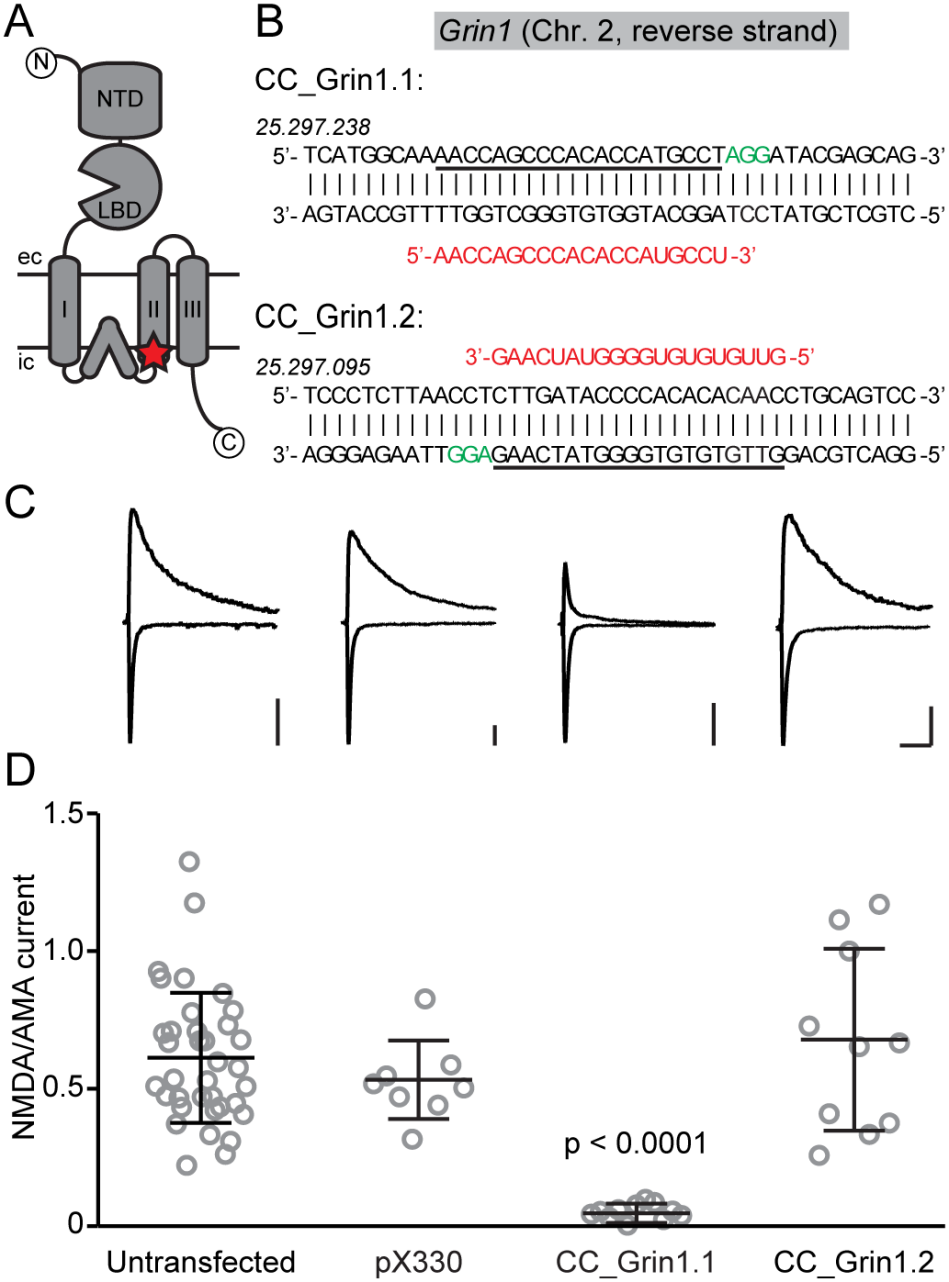
CRISPR/Cas9 mediated knock-down of NMDA receptors. (A) Schematic domain organization of a GluN1 subunit. Red star indicates location of the CC_Grin1.1 target sequence. Circled N/C indicate N-/C-terminus respectively; NTD: N-terminal binding domain; LBD: ligand binding domain; roman numbers indicate transmembrane domains 1–3, ec/ic: extracellular/intracellular. (B) Genomic sequences of CRISPR/Cas9 targets in GluN1. *Grin1* (encoding for mouse GluN1) is located on the reverse strand of chromosome 2, and the third transmembrane domain is encoded by exon 14. Target sequences are underlined, crRNA sequences are in red, PAM sequences in green. Numbers above DNA strand indicate chromosomal position. (C) Example traces of AMPAR currents (recorded at −70 mV, inward) and NMDAR currents (recorded at +40 mV, outward) from acute brain slices. From left to right: untransfected, pX330 (‘empty’ CRISPR/Cas9 without targeting sequence), CC_Grin1.1, CC_Grin1.2. Scale bars are 50 pA and 100 ms. (D) NMDAR/AMPAR current ratio for all cells. Untransfected control cells were interleaved with neighboring GFP-positive experimental cells. AMPAR current amplitude was measured as the peak inward current at −70 mV, while NMDAR current amplitude was measured 100 msec after stimulation at +40 mV, to avoid contamination with AMPARs. Bars indicate mean ± S.E.M., grey circles individual cells. Transfection with CC_Grin1.1 completely eliminated NMDAR currents (Kruskall-Wallis test, followed by Dunn’s test).

The genomic sequence surrounding the sequence encoding for the second transmembrane region of mouse GluN1 (±200 bp) was analyzed for potential CRISPR/Cas9 targets *in* *silico* [16]. The two sequences with the highest predicted ‘on-target score’, ‘CRISPR/Cas9 against *Grin1* .1/2’ (‘CC_Grin1.1’ and ‘CC_Grin1.2’, Figure 1B) were synthesized and subcloned into a vector (pX330, addgene plasmid #42230) containing the flanking gRNA sequences and a codon-optimized Cas9 [8].

### In-utero electroporations

Neuronal transfections were performed by *in-utero* electroporation of E15 wild-type C57BL/6J mice as described previously [17]. Briefly, an E15 pregnant mother was anesthetized using 2% isoflurane and injected with 0.1 mg/kg of buprenorphrine as anesthetic. Embryonic pups within the intact uterus were temporarily removed from the abdomen and injected into the left hemisphere with 1 µl of DNA mixture, containing the appropriate CRISPR-construct and soluble GFP (10∶1), using a ∼50 µm-diameter pipette sharply beveled at 15°–20° (Narishige, Japan), visually confirming the proper site of correct injection by mixing 0.005% fast green with the DNA. To target transfection to the hippocampus, the head of the embryonic pup was placed between paddles of tweezer electrodes (CUY21 electroporator, NEPA GENE, Japan), with the positive terminal covering the lateral surface of the right hemisphere and the negative terminal covering the left hemisphere. Each injected embryo was then subjected to 5×50 ms/35 V electric pulses. Following electroporation, the intact uterus containing the pups was returned to the abdomen, and the mother’s abdomen sutured shut. Recordings were made from transfected pups 14–20 days following birth.

### Biolistic transfection of organotypic slice cultures

Biolistic transfection of post-mitotic neurons was achieved using organotypic hippocampal slice cultures. Slice cultures were prepared from P6–8 wild-type Sprague-Dawley rats as described previously [18]. Slice cultures were maintained at 34°C with 5% CO_2_ on 30 mm Millicell Cell Culture inserts with 0.4 um pores (Millipore) in slice culture media containing: MEM (8.32 g/L), 20% heat-inactivated horse serum, 1 mM L-glutamine, 1 mM CaCl_2_, 2 mM MgCl_2_, insulin (1 mg/l), 13 mM D-Glucose, 5.2 mM NaHCO_3_, 30 mM HEPES, and 0.00125% ascorbic acid. Media was changed every 2–3 days. For biolistic transfection, a DNA mixture containing 50 µg of the CRISPR-construct and 30 µg of a GFP-expressing plasmid were precipitated onto 1 µm diameter gold particles, washed with ethanol, and coated onto the inside of Tefzel tubing (Bio-rad). After 2–3 days in vitro, slice cultures were shot with the DNA-coated gold cartridges using 100 PSI ultra-pure helium with a Helios GeneGun (BioRad). Transfected neurons were identified by the presence of both GFP epifluorescence and a gold particle in the neuronal cell body. Neurons that were GFP positive, but lacking a gold particle or visibly fused with neighboring cells were excluded from analysis.

### Electrophysiology

Whole-cell voltage clamp recordings were obtained from transfected hippocampal CA1 and CA3 pyramidal neurons as identified by GFP epifluorescence, as well as neighboring untransfected control cells. Initial target sequence validation was performed in neurons transfected via *in* *utero* electroporation. Transfected CA1 and CA3 neurons were recorded from 300 µm acute, horizontal brain slices of P14–20 mice, cut using a vibratome (Leica Biosystems) in a chilled sucrose cut solution containing (in mM): 2.5 KCl, 7 MgSO_4_, 1.25 NaH_2_PO_4_, 25 NaHCO_3_, 7 glucose, 210 sucrose, 1.3 ascorbic acid, and 3 sodium pyruvate. Following cutting, the slices recovered for 30 minutes in 34°C artificial cerebral spinal fluid (ACSF) containing (in mM): 119 NaCl, 2.5 KCl, 1 NaH_2_PO_4_, 26.2 NaHCO_3_, 11 glucose, 2.5 CaCl_2_, and 1.3 MgCl_2_. Both the cut solution and ACSF were bubbled with 95% O_2_/5% CO_2_ gas throughout dissection, slicing and recording. During recording, slices were perfused at a rate of 250 ml/hr with ACSF. 10 µM SR 95531 (Tocris) was added to the ACSF to block inhibition. For recordings from organotypic slice cultures, CaCl_2_ and MgCl_2_ concentration were both increased to 4 mM and 10 µM 2-chloroadensine (Tocris) added to prevent runaway excitation following stimulation. Synaptic responses were evoked using electrical stimulation of stratum radiatum from a tungsten bipolar electrode (FHC). To minimize polysynaptic responses, a cut was made at the border of CA3 and CA1 using a microscalpel (Electron Microscopy Sciences). Whole-cell recordings were made using 3–5 MΩ glass pipettes filled with internal solution containing (in mM): 135 CsMeSO_4_, 8 NaCl, 10 HEPES, 0.3 EGTA, 5 QX-314, 4 Mg-ATP, 0.3 Na-GTP, and 0.1 spermine, at 294 mOsm and pH 7.34. Whole-cell voltage clamp was performed using a Multiclamp 700B amplifier (Axon instruments) with a 3 kHz Bessel filter, digitized at 10 kHz using a National Instruments data acquisition board, and recorded using custom-written MatLab (MathWorks) software [19].

### Data analysis and statistics

Data were analyzed offline in Igor Pro (Wavemetrics). For each cell the average peak response at −70 mV holding potential was quantified from 10–20 sweeps as AMPAR mediated current, whereas an equal number of recordings at +40 mV where quantified at 100 ms post-stimulus as NMDAR current. These two values were used to calculate the NMDA/AMPA ratio, and for each cell the holding potential was switched several times back and forth during the recording, to account for potential changes in the evoked response over time. Different conditions were compared using non-parametric statistical tests, Mann-Whitney test for two groups and Kruskal–Wallis analysis of variance (ANOVA) followed by Dunn’s Multiple Comparison Test for multiple group comparisons.

## Results and Discussion

To test CRISPR/Cas9-mediated gene knockout in post-mitotic mouse neurons, we targeted *Grin1*, the gene encoding the GluN1 subunit of the N-methyl-D-aspartate-type glutamate receptor (NMDAR). This subunit is essential for NMDAR function [20], and the degree of GluN1 loss can be easily assayed by the amplitude of synaptic NMDAR currents [21]. Additionally, constitutive genomic deletion of *Grin1* is embryonic lethal [22], whereas a hypomorphic allele shows decreased NMDAR currents and severe neural dysfunction early in development [18]. A conditional GluN1 knockout mouse has been generated [23], and sparse transfection of Cre has been used to examine the effect of NMDAR deletion in individual hippocampal neurons [24], [25]. Thus, targeting GluN1 provides a well-characterized and robust experimental system to assay for the deletion of an essential protein in individual neurons of an otherwise unperturbed brain.

We identified two genomic CRISPR/Cas9 target sequences (see methods) within or near the region encoding for the second, pore-forming transmembrane domain of GluN1 (Figure 1A, B). ‘CC_Grin1.1’ is located on the sense strand of exon 14, spanning the sequence that encodes the beginning of the second transmembrane domain; ‘CC_Grin1.2’ is on the intron anti-sense strand between exon 13 and 14, about 130 bp upstream of CC_Grin1.1. The appropriate sequences were subcloned into a vector containing all other elements required for CRISPR/Cas9 mediated knock-down [8]. Identification of target sequences and the required cloning required less than three days, emphasizing the simplicity and effectiveness of this approach.

To functionally assay for NMDAR-deletion, CC_Grin1.1 and CC_Grin1.2 were transfected by *in-utero* electroporation into the hippocampus of wildtype mice, and a plasmid encoding GFP was co-transfected at a 1∶10 ratio. We measured the ratio of NMDAR-over AMPAR- (α-amino-3-hydroxy-5-methyl-4-isoxazolepropionic acid receptor) mediated currents at CA3 and CA1 pyramidal neurons (see methods), following electrical stimulation of dentate gyrus mossy fiber (MF) and CA3 Shaffer collaterals (SC) pathways, respectively. The ratio of the amplitudes of NMDAR and AMPAR mediated excitatory postsynaptic currents (EPSCs) in untransfected pyramidal neurons was 0.61±0.04 (n = 36), and transfection of a CRISPR/Cas9 construct lacking the targeting sequence (‘pX330’) had no effect on this ratio (0.53±0.05, n = 8). In contrast, cells transfected with CC_Grin1.1 showed complete loss of NMDAR currents (0.04±0.01, n = 12). Importantly, the functional loss of NMDAR by CC_Grin1.1 was observed in every cell tested, demonstrating effective CRISPR/Cas9 mediated knockdown with full penetrance and suggesting disruption of both genomic alleles. Using the same approach with CC_Grin1.2 did not show any effect (0.67±0.11, n = 10), and every cell tested showed robust NMDAR currents (Figure 1C). Together, the results demonstrate that CRISP/Cas9-mediated knockdown can be applied to neurons *in* *vivo*. However, since both CC_Grin1.1 and CC_Grin1.2 had similarly high predicted ‘on-target scores’ (94% and 92%, respectively) these results also show that any given target sequence needs to be confirmed individually.

**Figure 2:**
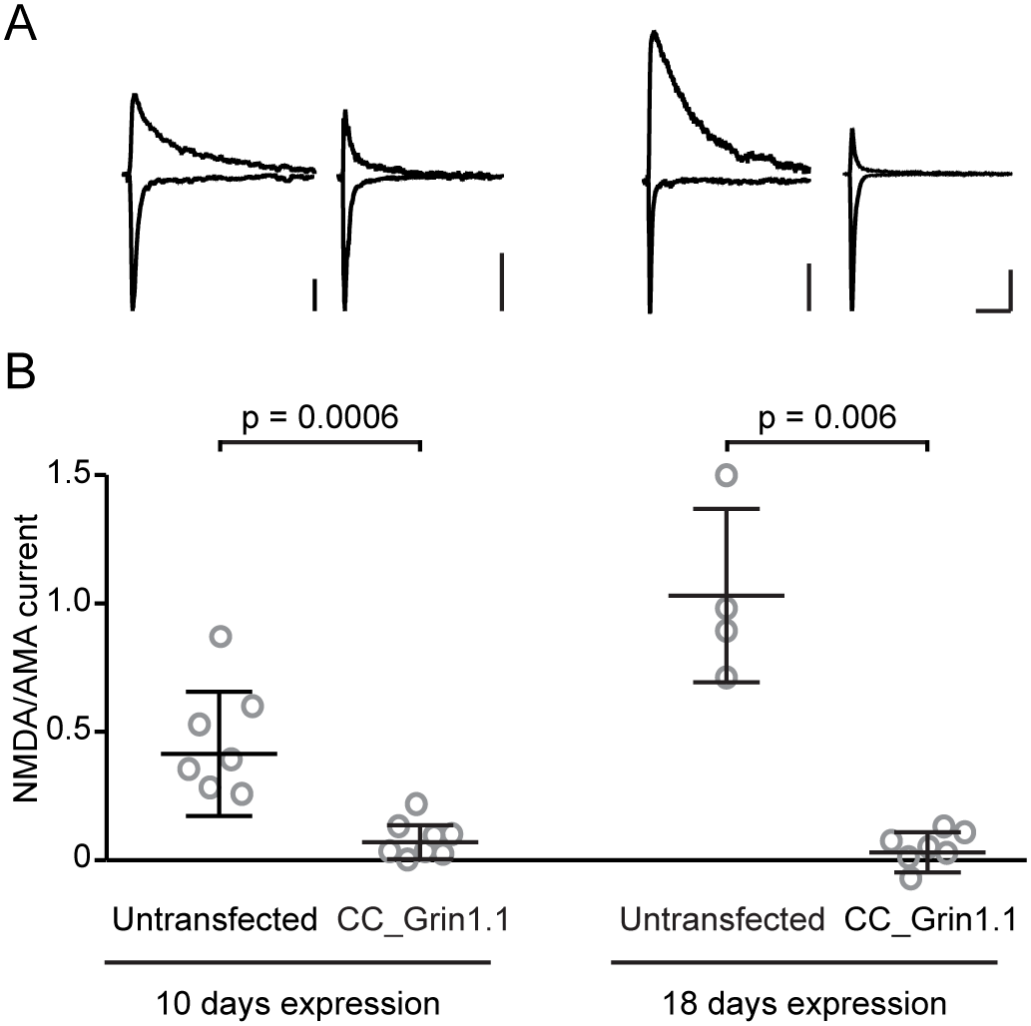
CRISPR/Cas9-mediated knock-down in postmitotic neurons. (A) Example traces of AMPAR currents (recorded at −70 mV, inward) and NMDAR currents (recorded at +40 mV, outward) from organotypic slice cultures. Shown are examples from untransfected cells and CC_Grin1.1 transfected cells following 10 days expression (left) and 18 days expression (right). Scale bars are 20 pA (left) or 50 pA (right) and 100 ms. (B) NMDAR/AMPAR current ratio for all cells as described before. Untransfected and CC_Grin1.1 transfected cells were compared individually for different time points, using the Mann-Whitney test.

Acute knockdown in the brain requires genetic modification in neurons – i.e. in post-mitotic cells. Since *in-utero* electroporation functions by transfecting mitotic precursor cells, we repeated our experiments in organotypic hippocampal slice cultures, where biolistic transfection occurs in post-mitotic neurons. The slice cultures were prepared from rat; however, the target sequence of CC_Grin1.1 is fully conserved between mouse and rat. Following 10 days of expression, CC_Grin1.1 significantly reduced NMDAR currents compared to untransfected neighboring control cells (Figure 2, left). However, some cells still showed a small residual NMDAR current, presumably due to slow turn-over of NMDAR protein [26]. We therefore repeated the experiment and expressed CC_Grin1.1 for 18 days, comparable to the expression time in acute slices following *in-utero* electroporation. This time window was enough to eliminate all NMDAR currents in CC_Grin1.1 transfected cells (Figure 2, right). These experiments in organotypic hippocampal slice culture demonstrate that complete CRISPR/Cas9-mediated knock-down can be achieved in acutely transfected post-mitotic neurons.

Altogether, our results show that the CRISPR/Cas9 system can be used to abolish proteins of interest from individual cells in the postnatal brain. The number of cells depends purely on the transfection efficiency, and this method is therefore ideally suited to prevent plastic adaption that can obscure knock-down effects. In comparison to other acute knock-down methods, CRISPR/Cas9-mediated knock-down is either more efficient and complete (e.g. RNA-interference based approaches [21]) or faster (generation of conditional knock-out mice and subsequent transfection with Cre), making it the ideal candidate for the investigation of neuronal function.

However, several technical challenges remain to be considered for use of CRISPR/Cas9 in neurons. A principal concern for the use of CRISP/Cas9 is that of off-target effects, which can be expected from a method that relies on a 20 bp recognition motif. Recent work has begun to address this problem [27], [28], and it remains the biggest challenge for future use of CRISP/Cas9. Guide RNA target-sequences will therefore not only need to be screened for efficacy, but selected to attempt to minimize off-target effects. Another important point for future use of CRISPR/Cas9 in the brain will be the development of effective gene delivery methods. Both *in-utero* electroporation and biolistic transfection of slice cultures allow for delivery of multiple large expression constructs, but are limited in terms of spatial and temporal specificity. The required CRISPR/Cas9 sequences are too large to be packaged into adeno-associated virus, the method of choice for genetic manipulation of mature neurons *in* *vivo* [29], and viral delivery will have to rely either on a two-virus system, or the use of alternative viral vectors that tolerate longer extrinsic DNA sequences, such as lentivirus.
